# Somatosensory lateral inhibition processes modulate motor response inhibition - an EEG source localization study

**DOI:** 10.1038/s41598-017-04887-z

**Published:** 2017-06-30

**Authors:** Julia Friedrich, Moritz Mückschel, Christian Beste

**Affiliations:** 1 Cognitive Neurophysiology, Department of Child and Adolescent Psychiatry, Faculty of Medicine, TU Dresden, Germany; 2 MS Centre Dresden, Faculty of Medicine of the TU Dresden, TU Dresden, Germany; 3 Experimental Neurobiology, National Institute of Mental Health, Klecany, Czech Republic

## Abstract

Motor inhibitory control is a central executive function, but only recently the importance of perceptual mechanisms for these processes has been focused. It is elusive whether basic mechanisms governing sensory perception affect motor inhibitory control. We examine whether sensory lateral inhibition (LI) processes modulate motor inhibitory control using a system neurophysiological approach combining EEG signal decomposition with source localization methods in a somatosensory GO/NOGO task. The results show that inter-individual variations in the strength of LI effects predominantly affect processes when information needs to be integrated between cerebral hemispheres. If information needs to be integrated between hemispheres, strong sensory suppression will lead to more impulsive errors. Importantly, the neurophysiological data suggest that not purely perceptual or motor processes are affected. Rather, LI affects the response selection level and modulates processes of stimulus categorization. This is associated with activity modulations in the posterior parietal cortex. The results suggest that when sensory suppression is high and when information needs to be integrated across hemispheres, these processes are less efficient, which likely leads to worse motor inhibitory control. The results show how basis principles modulating perceptual processes affect subsequent motor inhibitory control processes.

## Introduction

Response inhibition constitutes a central executive function^1^ that has extensively been investigated^2^. While it is obvious that to successfully deploy inhibitory control processes, perceptual and attentional processes are essential^3^, the role of perceptual processes during response inhibition processes has only recently been focused regarding motor inhibitory control^4–6^, but several lines of evidence suggest that perceptual processes predict response inhibition performance^4, 7^. While this underlines the importance to consider perceptual processes during motor inhibitory control, we are only at the verge to understand the role of perceptual processes during response inhibition. The relevance of core mechanisms governing perceptual processes has not been investigated for motor inhibition processes. One such basic principle modulating perceptual processes is lateral inhibition^8–10^. Lateral inhibition emerges when „a local network of inhibitory interneurons connects adjacent cortical neusrons so that firing of one cortical neuron tends to lead to inhibition of its neighbors”^8^. Since lateral inhibition mechanisms do therefore play a central role in the processing of incoming stimuli that have been shown to impact motor response inhibition^11^, we hypothesize that modulations of lateral inhibition mechanisms may well affect motor inhibitory control. Especially processing of stimuli within the somatosensory modality strongly depends on mechanisms of lateral inhibition^8, 12–14^. Because the somatosensory modality has been shown to be more powerful than the visual modality to trigger response inhibition processes^11^, it seems particularly suited to examine the effect of sensory lateral inhibition processes on motor inhibitory control in the current system neurophysiological study.

It is well-known that the distance between stimulation sites determines lateral inhibition effects with less distance leading to stronger lateral inhibition effects^12–16^. For example, it has been shown that simultaneous stimulation within one hand leads to attenuated perceptual processes as compared to the stimulation of both hands, suggesting that lateral inhibition effects are evident in the within-hand condition, but not in the between-hands condition^12^. We therefore compare response inhibition processes between one condition in which lateral inhibition can influence response inhibition (i.e. the within-hand condition) with a condition in which lateral inhibition effects do less occur due to the involvement of both hemispheres (i.e. the between-hands condition). We hypothesize that strong lateral inhibition processes are associated with better motor inhibitory control; i.e. response inhibition performance is better in the within-hand condition, than in the between-hands condition. This is because in response inhibition, the frequent “GO” stimulus leads to prepotent response tendencies^2, 17, 18^. Since lateral inhibition leads to a weaker representation of the stimuli, the activation of the associated prepotent response is weaker in the within-hand condition. This likely entails better response inhibition performance and would suggest that fundamental principles governing cortical stimulus representation affect subsequent motor response inhibition processes. To examine the strength of lateral inhibition on motor inhibitory control in more detail, we split the sample of participants in a “high suppression” (HS) and a “low suppression” (LS) group using a median split (refer methods section for details).

The underlying neurophysiological mechanisms can be examined using EEG recordings and the analysis of event-related potentials (ERPs) in combination with source localization analyses (sLORETA). Using ERPs, effects of lateral inhibition are likely to lead to modulations in the Nogo-N2 reflecting pre-motor processes like conflict monitoring or, to modulations in the Nogo-P3 reflecting the motor process of inhibition^19, 20^. However, ERP measures are usually composed of various amounts of signals from different brain regions and therefore reflect a mixture of processes and information being processed. To dissociate these different kinds of information we applied residue iteration decomposition (RIDE)^21–25^ on the EEG data and combine this with source localization analyses (i.e., sLORETA). RIDE decomposes EEG data on the basis of their timing variability properties. This decomposition reveals the S-c luster referring to processes related to the stimulus, the R-cluster depicting processes related to the response and the C-cluster representing intermediate processes between S and R (like stimulus evaluation and response selection)^23^. Using RIDE it has been shown that response inhibition mechanisms are reflected by the S-cluster and the C-cluster^22^. Both of these clusters may therefore be modulated by the experimental manipulation of lateral inhibition processes. However, since the critical aspect in response inhibition is the inhibition of a predominant transition of stimuli into a response, we hypothesize that it is especially likely that the C-cluster is modulated. This is because the C-cluster has been shown to reflect processes linking stimulus processing and responding^26^, and lateral inhibition may lead to a weaker representation of the stimuli that consequently entails a weaker activation of the associated prepotent response (see above). Moreover, the C-cluster has been shown to reflect mechanisms similar to the Nogo-P3 ERP component, which has frequently been linked to the inhibition of the motor response^20, 27, 28^. Such a result would suggest that lateral inhibition effects at the perceptual level spill over to the response selection level.

Regarding the functional neuroanatomical structures underlying modulations of response inhibition processes by lateral inhibition mechanisms, we expect that aside the usual response ‘inhibition network’ in the prefrontal cortex (i.e. the inferior and medial frontal cortex, supplementary motor area) also parietal structures become involved. This is due to specific stimuli applied and the emphasis on lateral inhibition processes. These parietal regions have already been shown to be involved in response inhibition processes using somatosensory stimuli^11^. Moreover, they are involved during sensorimotor integration^29, 30^, also during response inhibition when sensory information is difficult to categorize but essential for behavioral control^31–33^. This may be the case due to the effects of lateral inhibition.

## Results

### Behavioral data

The mixed effects ANOVA for GO hit rates revealed neither a main effect of “condition” [F(1,25) = 2.4; *p* = 0.134; *η²* = 0.088], nor a main effect “group” [F(1,25) = 0.88; *p* = 0.769; *η²* = 0.004], but a significant interaction of “condition × group” [F(1,25) = 5.42; *p* = 0.028; *η²* = 0.178]. However, post-hoc tests did not withstood Bonferroni-correction [all *t*(25) < 0.8; *p* > 0.435]. The analysis of reaction times (RTs) in GO trials revealed faster responses in the between-hands (458 ms ± 19) than in the within-hand condition (482 ms ± 20) [F(1,25) = 10.66; *p* = 0.003; *η²* = 0.299]. The interaction of “condition” × “group” [F(1,25) = 0.62; *p* = 0.437; *η²* = 0.024] as well as the between-subject factor “group” [F(1,25) = 2.23; *p* = 0.148; *η²* = 0.082] were not significant.

**Figure 1:**
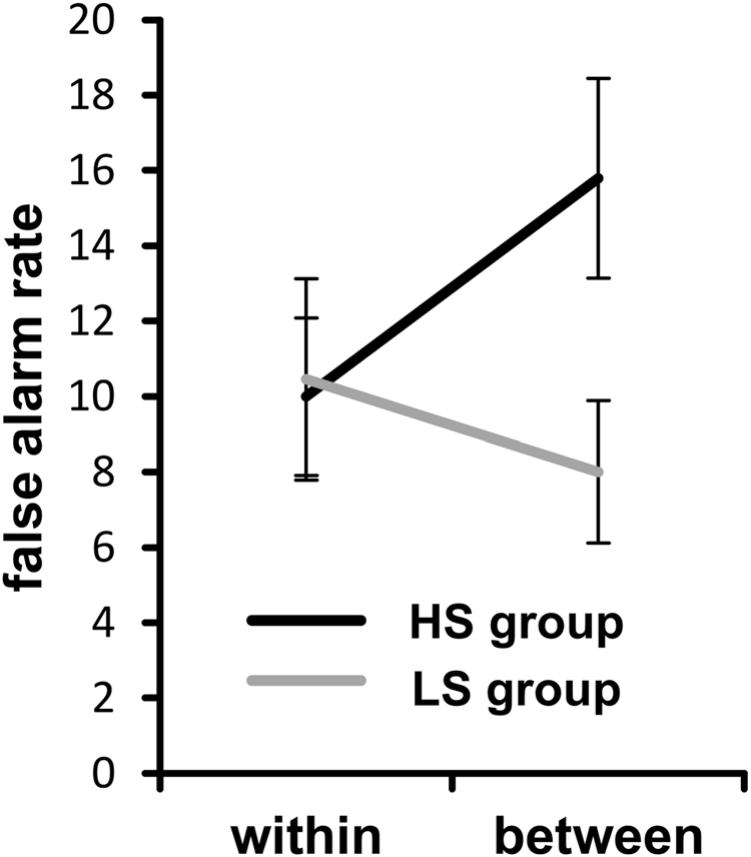
Behavioral data (false alarms rates) showing the interaction of condition × group. False alarm rate in the within-hand and between-hands condition is shown for the high (HS) and the low (LS) suppression group. Error bars represent the standard error of the mean (SEM).

Concerning false alarms in NOGO trials a main effect of “condition” revealed a significantly higher false alarm rate in the between-hands (12 ± 2) than in the within-hand condition (10 ± 2) [F(1,25) = 6.86; *p = *0.015; *η²* = 0.215]. Importantly, the interaction of “condition × group” reached significance [F(1,25) = 42.20; *p* < 0.001; *η²* = 0.628], which is shown in Fig. 1. Post-hoc independent-samples t-tests revealed that in the within-hand condition, the groups did not differ in their false alarm rates [*t*(25) = −0.14; *p* = 0.892]. In the between-hands condition the high suppression group had a significantly higher false alarm rate (16 ± 3) than the low suppression group (8 ± 2) [*t*(25) = 2.38; *p* = 0.025]. The between-subject factor “group” was not significant [F(1,25) = 1.27; *p* = 0.271; *η²* = 0.048].

### Standard event-related potentials (ERPs)

**Figure 2:**
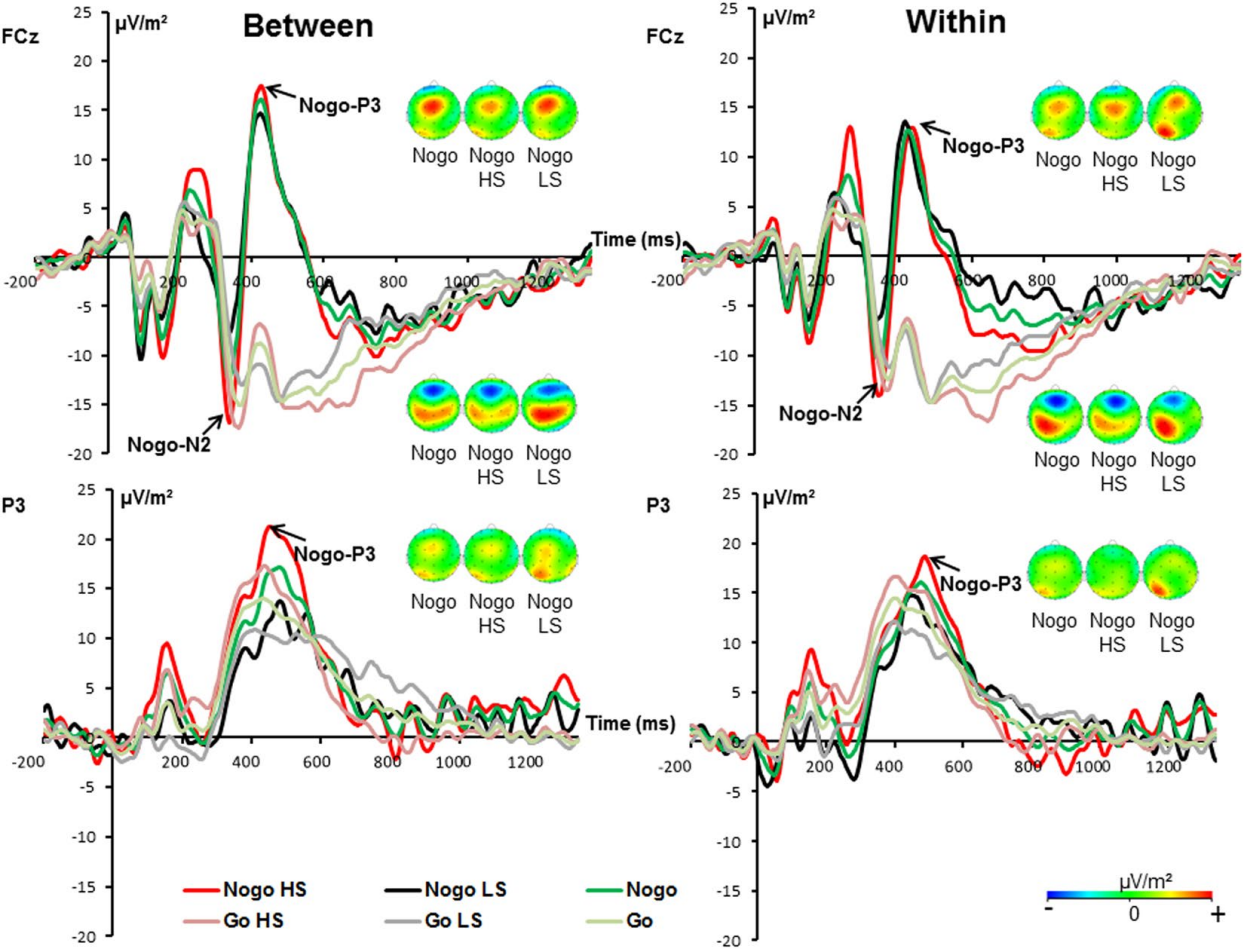
The P3 and N2 ERP components shown at electrode FCz (on top) and at electrode P3 (at the bottom) for the within-hand and the between-hands condition in GO and NOGO trials. In all plots time point 0 marks stimulus presentation. The different colors of the ERP traces correspond to the different experimental conditions as shown in the legend for GO and NOGO trials in the high (HS) and the low (LS) suppression group. The scalp topography plots represent the N2 and P3 in NOGO trials for the different conditions at the peak of the respective component. Red represents positive values and blue negative values.

The N2 and P3 ERP components are shown in Fig. 2. For the N2, the mixed effects ANOVA on the amplitudes revealed no significant main or interaction effect [all F ≤ 1.16; *p* ≥ 0.291]. As can be seen in the scalp topography plots, the P3 ERP was evident at electrode FCz and P3. Therefore, we added the factor “electrode” as an additional within-subject factor to statistical model. The mixed effects ANOVA revealed a main effect electrode [F(1,25) = 18.33; *p* < 0.001; *η²* = 0.423] showing that the P3 was larger at electrode P3 (3.21 ± 2.03) than at electrode FCz (14.47 ± 2.13). The main effect “GO/NOGO” [F(1,25) = 20.22; *p* < 0.001; *η²* = 0.447] showed that the P3 was larger in NOGO (14.90 ± 2.84) than in GO trials (2.79 ± 0.88). There was no main effect “group” [F(1,25) = 1.18; *p* = 0.287; *η²* = 0.045]. There was an interaction “electrode × GO/NOGO” [F(1,25) = 33.62; *p* < 0.001; *η²* = 0.574], showing that the P3 difference between GO and NOGO trials was larger at electrode FCz (22.23 ± 4.05) than at electrode P3 (1.93 ± 1.85) [*t*(25) = 5.90; *p* < 0.001]. No other main or interaction effects were significant [all F ≤ 0.82; *p* ≥ 0.374].

### Residue iteration decomposition (RIDE)

#### S-cluster

**Figure 3:**
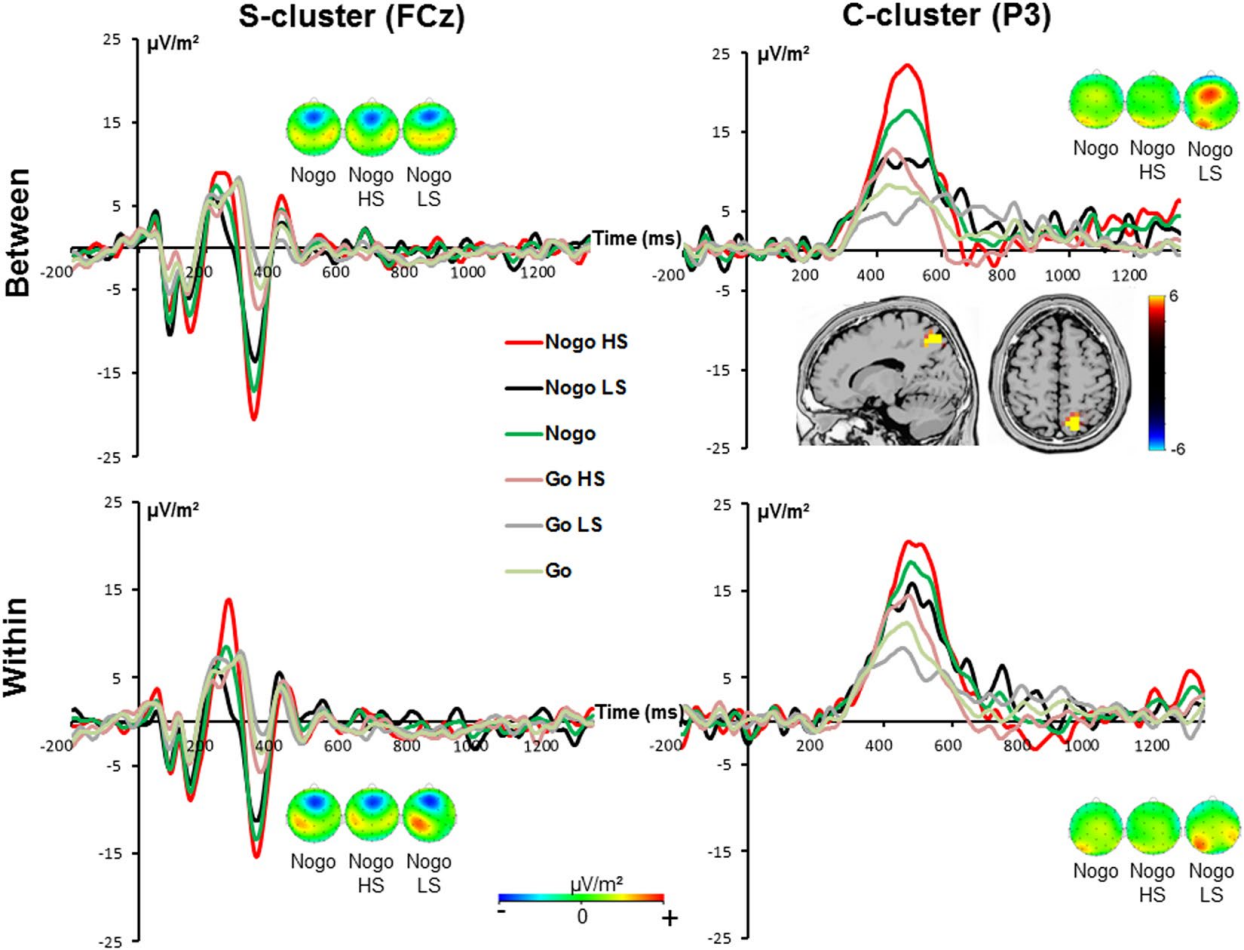
The S-cluster at electrode FCz (left) and the C-cluster at electrode P3 (right) is shown for the within-hand and the between-hands condition in GO and NOGO trials for the total sample (green and light green lines) as well as for the high (HS) and the low (LS) suppression group. In all plots time point 0 marks stimulus presentation. On the left the N2 component reflected in the S-cluster is shown as well as the scalp topography plots in NOGO trials. On the right the P3 component reflected in the C-cluster and the scalp topography plots in NOGO trials are illustrated. Red color represents positive values and blue color negative values. The sLORETA plot shows the source of the difference in the C-cluster modulation in NOGO trials in the high and the low suppression group. The sLORETA color scale shows the critical t-values (corrected for multiple comparisons).

The S-cluster was maximal at electrode FCz (refer Fig. 3). Applying a mixed effects ANOVA the main effect of “GO/NOGO” [F(1,25) = 24.13; *p* < 0.001; *η²* = 0.491] showed that the S-cluster amplitude was stronger in NOGO (−13.82 μV/m² ± 3.26) than in GO trials (−0.86 μV/m² ± 1.25). Furthermore, a main effect of “condition” was found [F(1,25) = 5.73; *p* = 0.024; *η²* = 0.187] with a larger amplitude in the between-hands (−8.57 μV/m² ± 2.18) than in the within-hand condition (−6.11 μV/m² ± 2.13). There were no further main or interaction effects [all F ≤ 1.92; *p* ≥ 0.178].

#### C-cluster

The C-cluster is also shown in Fig. 3 and revealed a parietal maximum around electrode P3. The mixed effects ANOVA revealed a main effect “GO/NOGO” [F(1,25) = 33.37; *p* < 0.001; *η²* = 0.572] showing that the C-cluster amplitude was larger in NOGO (17.58 μV/m² ± 2.67) than in GO trials (6.59 μV/m² ± 1.51). The main effect of “condition” was not significant [F(1,25) = 0.28; *p* = 0.601; *η²* = 0.011]. Importantly, there was a significant interaction “GO/NOGO × condition × group” [F(1,25) = 4.86; *p* = 0.037; *η²* = 0.163]. Subsequent ANOVAs revealed that there was an interaction “condition × group” for NOGO trials [F(1,25) = 4.3; *p* = 0.049; *η²* = 0.147], but not for GO trials [F(1,25) = 0.49; *p* = 0.486; *η²* = 0.020]. Post-hoc independent-samples t-tests revealed that this interaction originates from the difference in C-cluster amplitude between the high suppression group (23.4 μV/m² ± 2.79) and the low suppression group (10.55 μV/m² ± 5.94) in the between-hands condition [*t*(25) = 2.01; *p* = 0.028]. The sLORETA analysis revealed that activity differences were located in the right posterior parietal cortex (BA7). For the within-hand condition, the groups did not differ in the C-cluster amplitude [*t*(25) = 0.96; *p* = 0.344]. There were no other main or interaction effects in the C-cluster [all F ≤ 3.68; *p* ≥ 0.067].

#### R-cluster

**Figure 4:**
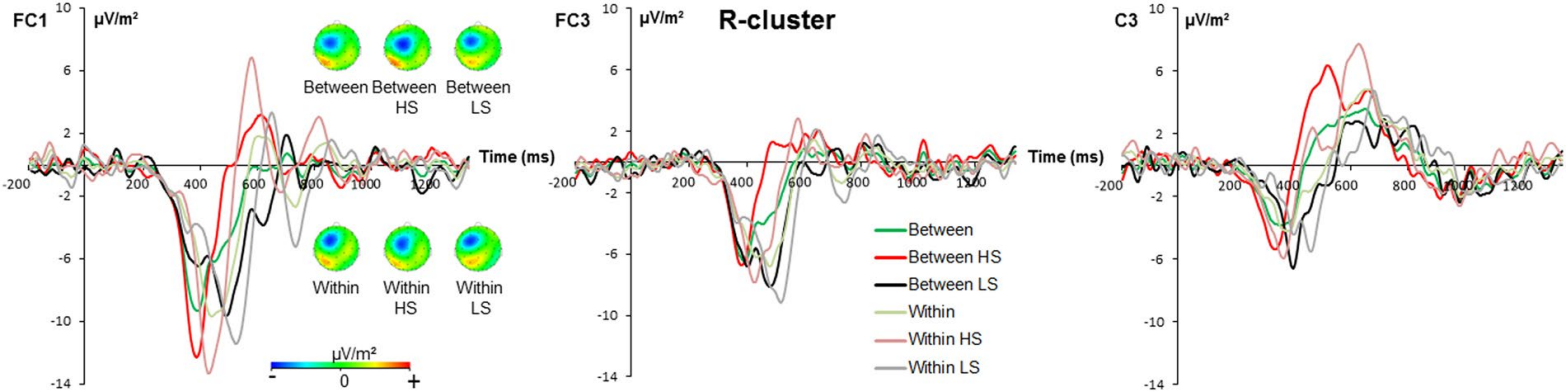
The R-cluster at electrodes FC1 (left), FC3 (middle) and C3 (right) is shown for the within-hand and the between-hands condition in GO trials for the total sample (green and light green lines) as well as for the high (HS) and the low (LS) suppression group. In all plots time point 0 marks stimulus presentation. The scalp topography plots represent the R-cluster in GO trials at the time point of response execution. Red color represents positive values and blue color negative values.

The R-cluster is shown in Fig. 4 for GO trials. As can be seen in Fig. 4 the R-cluster was evident at electrodes FC1, FC3, and C3, but strongest at electrode FC1. The analysis only revealed a main effect “electrode site” [F(2,50) = 5.79; *p* = 0.005; *η²* = 0.188] showing that the R-cluster was largest (most negative) at electrode FC1 (−9.21 μV/m² ± 2.02), followed by electrode FC3 (−6.17 μV/m² ± 1.37) and C3 (−3.73 μV/m² ± 0.90). Only electrode FC1 differed from electrode C3 (*p* = 0.021), no other differences between electrodes were obtained.

## Discussion

The current study was designed to investigate the effects of sensory lateral inhibition mechanisms on motor response inhibition processes. Therefore, a task using vibrotactile information as GO and NOGO stimuli was employed. To examine the system neurophysiological mechanisms modulated we combined ERP recordings with source localization analyses (sLORETA) in combination with temporal data decomposition methods (RIDE).

The results revealed that response inhibition performance was better in the within-hand than in the between-hands condition as reflected by a significantly lower false alarm rate. Due to the somatotopic organization of the primary somatosensory cortex, it is assumed that receptive fields of neighboring fingers overlap^12, 15, 16^, resulting in a reduction of neuronal stimulus representation caused by lateral inhibition when nearby somatotopic regions are stimulated^12, 13^. This suggests that it is easier to withhold a prepotent response under the influence of lateral inhibition, compared to a condition where lateral inhibition processes are unlikely to occur, since simultaneously delivered information is processed in different hemispheres. The possible reason for this may be as follows. A high rate of GO stimuli leads to a strong response tendency^18^, which may be particularly so in case of somatosensory stimuli, because of strong structural connections of the primary somatosensory and motor cortices^34^. Since lateral inhibition effects may attenuate cortical representation of sensory stimuli, the activation of a response and the response tendency is also weaker, making it easier to withhold the dominant response. Consequently, less false alarms occur. This interpretation is underlined by RT data in GO trials, which are slower in the with-hand condition. Yet, it is important to note that this main effect of condition and hence its interpretation has to be handled with caution since there is a higher order interaction with the factor “group”. Notably, lateral inhibition does not affect the execution of an action per se but specifically unfolds its beneficial effects when motor plans have to be revised, because no condition effects were observed in GO trials.

However, to maximize the lateral inhibition effect in order to examine the neurophysiological processes being modulated by the effects of lateral inhibition, participants were assigned to the low or high suppression group. A significant interaction of group and condition was obtained. Interestingly, no group differences were evident in the within-hand condition. In the between-hands condition the high suppression group showed a higher false alarm rate than the low suppression group. This pattern of results suggests that in conditions where lateral inhibition is possible (i.e. the within-hand condition), even small lateral inhibition effects are sufficient to reach the performance level of the group showing marked lateral inhibition effects. Obviously, the beneficial effect of lateral inhibition on response inhibition is so strong that even a small degree is enough to notably enhance performance. Yet, in the condition in which the advantage of lateral inhibition cannot be used (i.e. in the between-hands condition), the inhibition performance of the group showing largest lateral inhibition effects significantly drops. The neurophysiological data provide insights to the cognitive subprocesses related to this effect.

For the neurophysiological data, no effects paralleling the behavioral results were found on the basis of traditional ERPs, but only after applying RIDE. RIDE is a temporal decomposition method that reduces intra-individual variability in the data^35^. Of note, this decomposition reveals three clusters of components with dissociable functional relevance^23^: the S-cluster refers to processes related to the stimulus (like perception and attention), the R-cluster refers to processes related to the response (like motor preparation and execution) and the C-cluster refers to intermediate processes between S and R (like stimulus evaluation and response selection)^23^. Interestingly, an effect paralleling the obtained interaction was not found for the S-cluster. Even though lateral inhibition effects are a mechanism well-described for the level of perceptual processing^8, 15, 16^, the RIDE data show that modulations at the stimulus processing level do not underlie the effects of lateral inhibition processes during motor inhibitory control. Since no modulations paralleling the behavioral data were obtained for the R-cluster, also pure motor processes do not seem to be important. Rather, the results suggest that effects of lateral inhibition are protracted to the response selection level, since an interaction effect paralleling the behavioral data was found in the C-cluster. There were significant differences in C-cluster amplitudes for the NOGO but not the GO trials. The C-cluster amplitude in NOGO trials was larger in the high than in the low suppression group and consequently higher in the group also showing more false alarms. The C-cluster was maximal in the P3 time window. Usually a higher P3 in NOGO trials is associated with better behavioral performance and in these cases the medial frontal cortical areas are involved^20^. The C-cluster, however, has been suggested to reflect processes related to stimulus-response transition^26^. In case of the current study, amplitude modulations in the C-cluster between the low and high suppression group were related to activation differences in the right posterior parietal cortex (PPC) activity (BA 7) as indicated by the sLORETA analysis. The posterior parietal cortex has also been linked to sensorimotor integration and hence the transformation of incoming sensory information into appropriate behavior^29^. Yet, opposed to medial frontal areas, the superior parietal cortex (BA7) has less frequently been reported to be involved in response inhibition^17, 32, 36, 37^. However, BA7 is involved in response inhibition whenever information is complex and probably difficult to categorize but essential for behavioral control^31–33^. This is also likely the case in the current study, and especially so in the between-hands condition, because information from both hands need to be integrated. The results therefore suggest that in the high suppression group these processes are intensified and less efficient than the low suppression group. This relative lack of efficacy to categorize stimuli essential for behavioral control, compared to the low suppression group, may lead to the higher false alarm rate in the high suppression group. Corroborating this interpretation, it has already been shown that if stimuli are presented to a hemisphere that is not optimized for processing this information due to functional cerebral asymmetries, the efficacy of response inhibition processes is compromised^32^. Due to the effect of perceptual processes on motor inhibitory control described in the current study, one can only speculate about the potential relevance of structural connectivities between somatosensory and prefrontal areas. It has been shown that the primary, but also the secondary somatosensory cortex is directly connected to areas that are part of a motor inhibitory network^2, 38, 39^, although the connections between the SI and prefrontal cortex are more pronounced^34, 40^. The SII, however, is engaged in the cognitive processing of tactile information^41^. So, future studies should be designed to assess the contribution of each area to response inhibition considering the fact that both areas are involved in the processing of different kinds of vibrotactile stimuli^42, 43^. Furthermore, the current study investigated sensorimotor integration processes on a correlative level. One possibility to evaluate whether there is a causal relationship between somatosensory and motor areas is the application of transcranial direct current stimulation which has already been shown to modulate activity in the somatosensory cortex^44–46^.

To summarize, the study examined in how far lateral inhibition effects modulate motor inhibitory control processes. Motor inhibitory control is better when (somatosensory) stimuli, which are presented in parallel, are processed in the same hemisphere. This likely reflects the result of lateral inhibition processes. However, inter-individual variations in the strength of lateral inhibition effects do predominantly affect processes when information needs to be integrated between cerebral hemispheres. If information needs to be integrated between hemispheres, strong sensory suppression will lead to more impulsive errors. Importantly, the neurophysiological data suggest that not purely perceptual or motor processes are affected. Rather, effects of lateral inhibition are protracted to the response selection level and seem to affect processes of stimulus categorization associated with activity modulations in the posterior parietal cortex (PPC, BA7). The results suggest that when sensory suppression is high and when information needs to be integrated across hemispheres, these processes are intensified and less efficient, which likely leads to worse motor inhibitory control. The results show how basis principles modulating perceptual processes affect subsequent motor inhibitory control processes.

## Materials and Methods

### Participants

N = 27 participants (17 females) took part in this study. Their age ranged from 19 to 30 years (mean age = 24.41; SEM = 0.58). They were all right-handed and reported no psychiatric or neurological disorders. Prior to testing, written informed consent was obtained from the subjects. The study and experimental protocols were approved by the institutional review board of the Medical faculty of the TU Dresden. All methods were performed in accordance with the relevant guidelines and regulations. To analyze the effects of lateral inhibition, i.e. to maximize the effects of lateral inhibition in the statistical analyses, the difference between false alarms in the between-hands and the within-hand condition was calculated for each participant. Using this calculated parameter, a median split of the sample was conducted. Participants with values below or equal to the median were assigned to the “high suppression group” (N = 14), whereas participants with values above this median were assigned to the “low suppression group” (N = 13).

### Procedure and task

**Figure 5:**
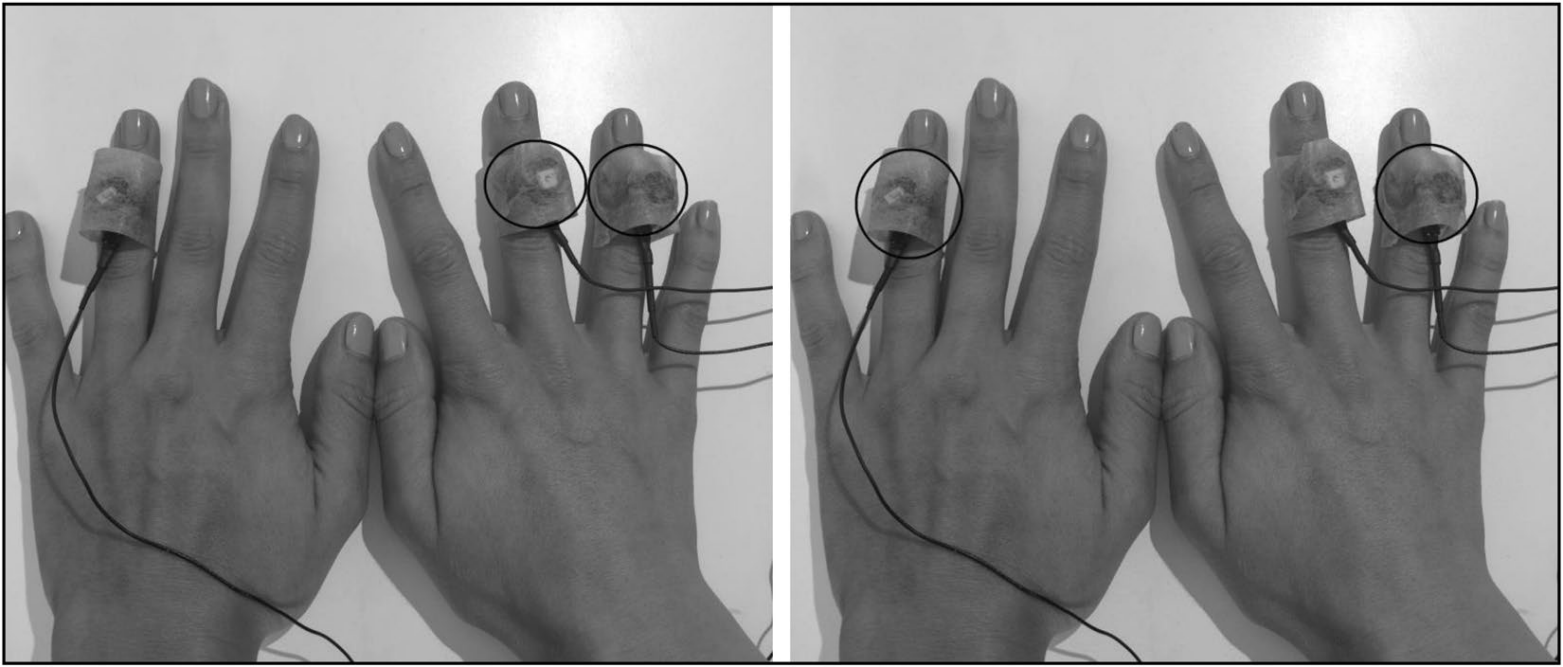
Illustration of the experimental setup. Active stimulators in the respective condition are circled in black. The left part shows the within-hand condition, the right part the between-hands condition. In the within-hand condition, GO and NOGO stimuli are simultaneously delivered to the ring and middle finger of the right hand. In the between-hands condition, the ring fingers of both hands were simultaneously stimulated using GO and NOGO stimuli.

To investigate the effects of lateral inhibition on response inhibition processes a tactile Go/Nogo task was employed. Vibrotactile stimuli were presented using small electromagnetic stimulators (Dancer Design; for more detailed information see http://www.dancerdesign.co.uk) that were connected to the “main module” of the stimulation device (Neurocore; http://www.neurocore.de/). The experimental setup is shown in Fig. 5. Either both hands, or just the right hand were stimulated. In the between-hands condition (stimulation of both hands) the tactile stimulators on the left and the right ring fingers vibrated simultaneously (but always with the same frequency, so GO and NOGO stimulation was not mixed up). The same fingers on both hands were used to ensure that the neuronal representation of the stimuli is equal in both hemispheres. In the between-hands condition, sensory input is evident in the left and right hemisphere foreclosing an effect of intra-hemispheric lateral inhibition^12, 14^. In the within-hand condition (right hand stimulation) the middle and ring finger of the right hand were stimulated simultaneously (with corresponding frequencies). Simultaneous stimulation of separate sites at the same hand causes lateral inhibition due to the activation of overlapping receptive fields^12–14^. These fingers were chosen, because the middle and ring finger stimulation is more effective than for example the middle and small finger stimulation because in case stimulation sites are closer to another interference is more pronounced than with greater distance between them^14, 16^. Lateral inhibition is not relevant in the between-hands condition since only if the same stimulus (GO or NOGO stimulus) is simultaneously delivered to two fingers with overlapping receptive fields lateral inhibition can occur. The receptive fields of the right and left hand ring fingers cannot overlap because they are in differed hemispheres.

Before the experiment started the vibrotactile stimulators were attached to the participants‘ fingers. A vibration of 15 Hz lasting 150 ms served as GO stimulus, whereas the 40 Hz stimulation of equal duration was set as NOGO stimulus (note that the respective stimulus was always applied to both fingers). The participants were acquainted with these stimuli, before the experiment was started. During the experiment, participants were asked to look at a white fixation cross in the middle of a black screen. This was done to prevent the subjects from closing their eyes and getting tired or moving their eyes excessively. In order to prevent the processing of any auditory feedback generated by the conductors, the subjects wore earplugs. The participants were asked to respond as fast as possible to the occurrence of the slow frequency by pressing a button with the right hand and to avoid responses to the fast frequency. In sum, 832 trials assigned to 4 blocks were presented. The trials were pseudorandomized to avoid sequence effects in the stimulation. The inter-trial interval was jittered between 700 and 1100 ms so that stimulus onsets were not predictable. To foster a premature response tendency, GO stimuli were presented in 70% of trials and a NOGO stimulus in 30% of trials. Conditions were blocked in order to prevent the participants from getting confused by constantly changing stimulation sites across the between-hands and within-hand conditions. Every subject received two within-hand and two between-hands blocks with breaks in between and the block sequence was counterbalanced across participants. With “A” representing the within-hand condition and “B” the between-hands condition, subjects either received the ABBA or BAAB sequence. The described block sequences were employed to reduce expectancy effects in regard to the upcoming block.

### EEG recording

60 passive Ag/AgCl ring electrodes at equidistant positions connected to a QuickAmp amplifier (BrainProducts Inc.) were used to record EEG data. The ground and reference electrode were placed at coordinates theta = 58, phi = 78 and theta = 90, phi = 90, respectively. The sampling rate was 500 Hz and the impedances were kept below 5 kΩ. After acquisition of data the sampling rate was changed to 256 Hz and an IIR band-pass filter from 0.5 Hz to 20 Hz using a slope of 48 db/oct as well as a notch filter at 50 Hz was applied to the un-epoched data set. Infrequent (technical or muscular) artifacts were discarded during a first manual raw data inspection. Afterwards, an independent component analysis was applied (ICA; infomax algorithm) to detect blinks, eye movements or pulse artifacts. ICA components revealing horizontal and vertical eye movements, blinks and pulse artifacts were manually rejected. The ICA was run for all blocks combined. Subsequently, the data was segmented in GO and NOGO trials for the between-hands and the within-hand condition according to the presentation of the stimuli. A segment started −200 ms before stimulus presentation and ended 1300 ms after it. Only trials answered correctly were included in analysis. In GO trials, the correct answer was defined as pressing the specified button in the time interval between target presentation and up to 900 ms after it. In NOGO trials, no registration of button press was defined as correct. Then, an automated artifact rejection procedure excluded trials in which a maximal value difference of 200 μV in a 200 ms period was exceeded. Furthermore, trials with amplitudes below −200 μV and above 200 μV as well as amplitudes below 0.5 μV in a 100 ms interval were rejected. To eliminate the reference potential from the data and to re-reference the data, we applied a current source density (CSD) transformation which results in values for amplitudes in μV/m^2^. We used 4 splines and 10 polynominals. CSDs work as a spatial filter^47, 48^, which accentuates electrode sites and makes it easier to identify electrode sites that best reflect relevant neuronal activity. This was followed by a baseline correction from −200 ms to 0 (time point of stimulus presentation). Finally, trials in the different conditions were averaged on a single-subject level. Grand averages for the two conditions (within/between) for GO and NOGO trials were computed separately for both groups. Scalp distribution and the course of averaged data was inspected visually before decisions about the to be analyzed components and electrodes of interest were made. The N2 and P3 components were evident at electrode FCz. In GO and NOGO trials the time interval from 305 to 315 ms was chosen in the high suppression group in the within-hand and between-hands condition. Correspondingly, this was also done for the low suppression group. The P3 was quantified in the range from 395 to 405 ms in the high and low suppression group for both conditions in GO and NOGO trials. Comparing the difference waves (calculated on the basis of NOGO trials in the within-hand and between-hands condition) of the low and high suppression group revealed that the P3 ERP was also evident at P3 electrode. Therefore, the mean amplitude of the P3 component at electrode P3 for NOGO trials was computed for the within-hand and between-hands conditions. Correspondingly, this was also done for the GO trials. In both groups, the mean P3 amplitude was quantified based on the time interval from 415 to 425 ms after stimulus onset.

### Residue iteration decomposition (RIDE)

Based on previous work^23, 26^ the RIDE toolbox (available on http://cns.hkbu.edu.hk/RIDE.htm) was employed to conduct RIDE analysis using MATLAB (MATLAB 12.0; Mathworks Inc.). In the following the RIDE method is briefly described. Further information concerning the RIDE decomposition algorithm are stated in Ouyang *et al*.^21^. RIDE decomposes ERP components applying *L1*-norm minimization (i.e., obtaining median waveforms) and therefore minimizes residual error due to temporal variability in single trials^21^. RIDE decomposition is based on latency variability and is applied to each electrode separately regardless of scalp distributions or waveforms^35^. Therefore, the conducted CSD transformation does not affect the results. The decomposition of the ERPs into S- and R-cluster (components) is based on stimulus onsets and response times, respectively, while the time markers for deriving the C-cluster are estimated and iteratively improved. It is assumed that there is a C-cluster with variable latency over single trials which is defined as being fully locked neither to stimulus onsets nor to reaction times, and that the latency of this C-cluster may be initially estimated in each single trial as reflecting some global waveform^26^. Based on the information given by the estimated latency of the C-cluster, by the time markers of stimulus onsets and by reaction times RIDE uses a self-optimized iteration scheme for latency estimation through which the latency estimation of the C-cluster is improved. The RIDE algorithm uses a time window function to extract the waveform of each RIDE component. Each time window is assumed to cover the range within which each component is supposed to occur and the specific values should be adjusted to fit the data on application^21^. For the current study this was from −200 to 800 ms for the S-cluster, from 200–800 ms for the C-cluster and 300 ms around the response trigger (−300 to 300 ms) for the R-cluster in GO trials. For further details on the method see refs 21 and 23.

In correspondence to the steps described above for the original ERPs the computation of the RIDE components was preceded by the visual examination of difference waves at relevant electrodes. Again, groups were compared in the within-hand and between-hands condition calculated on the basis of NOGO trials. The S-cluster was maximal at electrode FCz. Mean amplitudes of the S-cluster were computed in the interval from 305 to 325 ms for GO and NOGO trials in both conditions. The C-cluster was maximal at the parietal electrode P3. The mean amplitude was calculated for GO and NOGO trials in the within-hand as well as between-hands condition. For the GO trials the time interval from 520 to 530 ms was chosen for both groups and conditions. In NOGO trials within the between-hands condition, the period from 425 to 435 ms was used for quantification in the low suppression group. In the high suppression group the interval ranged from 465 to 475 ms. For both groups the C-cluster was calculated between 450 and 460 ms in the within-hand condition. For GO trials, the R-cluster was quantified in amplitude in the time window corresponding to RT in the between-hands and the within-hand conditions in the high and low suppression group; i.e. the negativities between 360 ms to 580 ms were quantified.

### Source localization analysis

For the source localization analysis sLORETA (standardized low resolution brain electromagnetic tomography)^49^ was used. As a basis for the source localization analysis we used the estimated RIDE clusters. sLORETA provides a single solution to the inverse problem^49–51^. For sLORETA, the intracerebral volume is partitioned into 6239 voxels at 5 mm spatial resolution. Then, the standardized current density at each voxel is calculated in a realistic head model^52^ based on the MNI152 template^53^. It has been mathematically proven that sLORETA provides reliable results without a localization bias^51^. Moreover, there is evidence from EEG/fMRI and neuronavigated EEG/TMS studies underlining the validity of the sources estimated using sLORETA^51, 54^. The voxel-based sLORETA images were compared across conditions (NOGO trials in the within-hand vs. between-hands condition) using the sLORETA-built-in voxel-wise randomization tests with 2000 permutations, based on statistical nonparametric mapping (SnPM). Voxels with significant differences (p < 0.01, corrected for multiple comparisons) between contrasted conditions were located in the MNI-brain.

### Statistical Analysis

Behavioral data, i.e. hit rates in GO trials and hit reaction times as well as false alarms in NOGO trials were examined by means of mixed effects ANOVAs. “Group” (low/high suppression group) was set as between-subject factor and “condition” (within/between) as within-subject factor. Mixed effects ANOVAs were also applied for neurophysiological data so that “group” constituted the between-subject factor and “condition” and “trial type” (GO/NOGO) the within-subject factors. Greenhouse-Geisser correction was applied for all tests. Bonferroni correction was conducted for all post-hoc tests. All variables were normal distributed as indicated by Kolmogorov Smirnov tests (all z < 0.9; p > 0.3). Mean values and the standard error of the mean (SEM) are given for the descriptive statistics.

### Data availability statement

The datasets generated during and/or analysed during the current study are available from the corresponding author on reasonable request
